# Euglycemic diabetic ketoacidosis associated with sodium-glucose cotransporter-2 inhibitor use: a case report and review of the literature

**DOI:** 10.1186/s12245-019-0240-0

**Published:** 2019-09-05

**Authors:** Alexis Diaz-Ramos, Wesley Eilbert, Diego Marquez

**Affiliations:** 1 Department of Emergency Medicine, University of Illinois College of Medicine at Chicago, 1819 West Polk St. 469 COME, Chicago, IL 60612 USA; 2 University of Illinois College of Medicine at Chicago, 1853 West Polk St. MC 785, Chicago, IL 60612 USA

**Keywords:** Euglycemic diabetic ketoacidosis, Euglycemic DKA, Sodium-glucose cotransporter-2 inhibitor diabetic ketoacidosis, Sodium-glucose cotransporter-2 inhibitor euglycemic diabetic ketoacidosis, SGLT2 inhibitor DKA, SGLT2 inhibitor euglycemic DKA

## Abstract

**Background:**

The sodium-glucose cotransporter-2 (SGLT2) inhibitors are the newest class of anti-hyperglycemic medications used in the treatment of diabetes mellitus. Their increasing use has been driven by their apparent cardiovascular and renal benefits. They have been associated with a small but significantly increased risk of diabetic ketoacidosis (DKA). Many of the cases of DKA associated with SGLT2 inhibitor use present with normal or minimally elevated serum glucose levels, often delaying the diagnosis.

**Case presentation:**

A 44-year-old woman with diabetes mellitus presented to our emergency department complaining of 3 days of generalized weakness. The SGLT2 inhibitor canagliflozin had been added to her medication regimen 4 weeks earlier, and she had stopped using insulin 2 weeks prior to presentation. Laboratory evaluation revealed a metabolic acidosis with an elevated anion gap and the presence of serum acetone, despite a minimally elevated serum glucose of 163 mg/dL. The patient was treated for euglycemic DKA with intravenous infusions of insulin and dextrose, with resolution of her symptoms in 3 days.

**Conclusions:**

The SGLT2 inhibitors are a novel class of anti-hyperglycemic medications that are being used with increasing frequency in the treatment of diabetes mellitus. They are associated with a small but significantly increased risk of DKA. Many of the patients presenting with DKA associated with SGLT2 inhibitor use will have normal or minimally elevated serum glucose levels. This unusual presentation of DKA can be diagnostically challenging.

## Background

The American Diabetes Association has defined diabetic ketoacidosis (DKA) with the following diagnostic criteria: metabolic acidosis (arterial pH < 7.3 and sodium bicarbonate < 18 mmol/L), ketosis (ketonemia or ketonuria), and hyperglycemia (serum glucose > 250 mg/dL) [1]. In 1973, Munro et al. published the first case series of patients with DKA despite normal or minimally elevated serum glucose levels and describe the condition as euglycemic diabetic ketoacidosis (EDKA) [2]. Further investigations have revealed that up to 7% of reported DKA cases have a serum glucose less than 250 mg/dL [3]. Conditions associated with EDKA include pregnancy, heavy alcohol consumption, pancreatitis, decreased caloric intake, insulin pump use, chronic liver disease, and glycogen storage disorders [3, 4].

The sodium-glucose cotransporter-2 (SGLT2) inhibitors are a new class of anti-hyperglycemic medications first introduced in 2013 [5]. Canagliflozin, dapagliflozin, and empagliflozin are the most widely used medications in this class. These medications lower serum glucose levels by the novel mechanism of increasing glucose clearance in the urine, making them unlikely to cause hypoglycemia. Within 3 years of their use in global markets, both the US Food and Drug Administration (US FDA) and the European Medicines Agency issued warnings that SGLT2 inhibitor use may predispose to DKA [6, 7]. Blau et al. reviewed the US FDA Adverse Event Reporting System regarding DKA associated with SGLT2 inhibitor use and found that 71% of the reported cases were EDKA [8].

In this report, we describe a case of EDKA associated with SGLT2 inhibitor use. Its pathophysiology, keys to diagnosis, and treatment will be discussed. Also, a review of the recent literature on this new, rare condition will be presented.

## Case presentation

A 44-year-old Hispanic woman presented to our emergency department (ED) complaining of generalized weakness for the previous 3 days. She denied having associated fever, vomiting, diarrhea, or shortness of breath. She denied experiencing any pain. Her past medical history was remarkable for diabetes mellitus (DM), for which she was taking metformin 500 mg twice daily, sitagliptin 100 mg twice daily, and canagliflozin 100 mg daily. The canagliflozin had been added to her medication regimen approximately 4 weeks earlier in the hopes of assisting her attempts to lose weight. The patient had been taking insulin as well up until 2 weeks before ED presentation, when it was discontinued by her endocrinologist who felt it was no longer needed for glycemic control.

On ED presentation, the patient was afebrile (36.7 °C), with a pulse rate of 79 bpm, blood pressure of 115/77 mmHg, and a respiratory rate of 18 bpm. She appeared well and in no distress. Her physical examination was notable for a normal neurologic exam including mental status, gait, and strength testing. Laboratory testing revealed a serum glucose of 163 mg/dL, a low serum bicarbonate of 14 mmol/L (reference range 21–31 mmol/L), and an elevated anion gap of 18 mmol/L (reference range 3.6–11.0 mmol/L). The remainder of her serum electrolytes as well as her blood urea nitrogen and creatinine was within normal limits. A serum lactate level was within the range of normal. Ketones were present in the urine, and acetone was present in the serum. Venous blood gas analysis found a pH of 7.27 with a PCO2 of 29 mm/Hg.

After an initial bolus of intravenous (IV) 0.9% normal saline, the patient was started on a continuous IV infusion of insulin with a second infusion of 5% dextrose and sterile water added to prevent hypoglycemia. The patient was admitted to the intensive care unit for treatment of EDKA. All further doses of canagliflozin were withheld. On hospital day 3, the patient’s ketosis and acidosis had resolved and she was discharged with insulin glargine, metformin, and sitagliptin for future control of her serum glucose levels.

## Discussion

The SGLT2 inhibitors are currently recommended as second-line medications in the treatment of type 2 DM, though they may be used as the primary medication for this purpose [9]. They are also used off-label in the treatment of type 1 DM [10]. Their increasing use has been bolstered by recent studies that suggest they provide some protection against major adverse cardiovascular events and death, reduce hospitalization for heart failure, and slow the progression of chronic kidney disease in type 2 DM [11, 12]. They also appear to be associated with modest reductions in weight and systolic blood pressure [13].

**Fig. 1:**
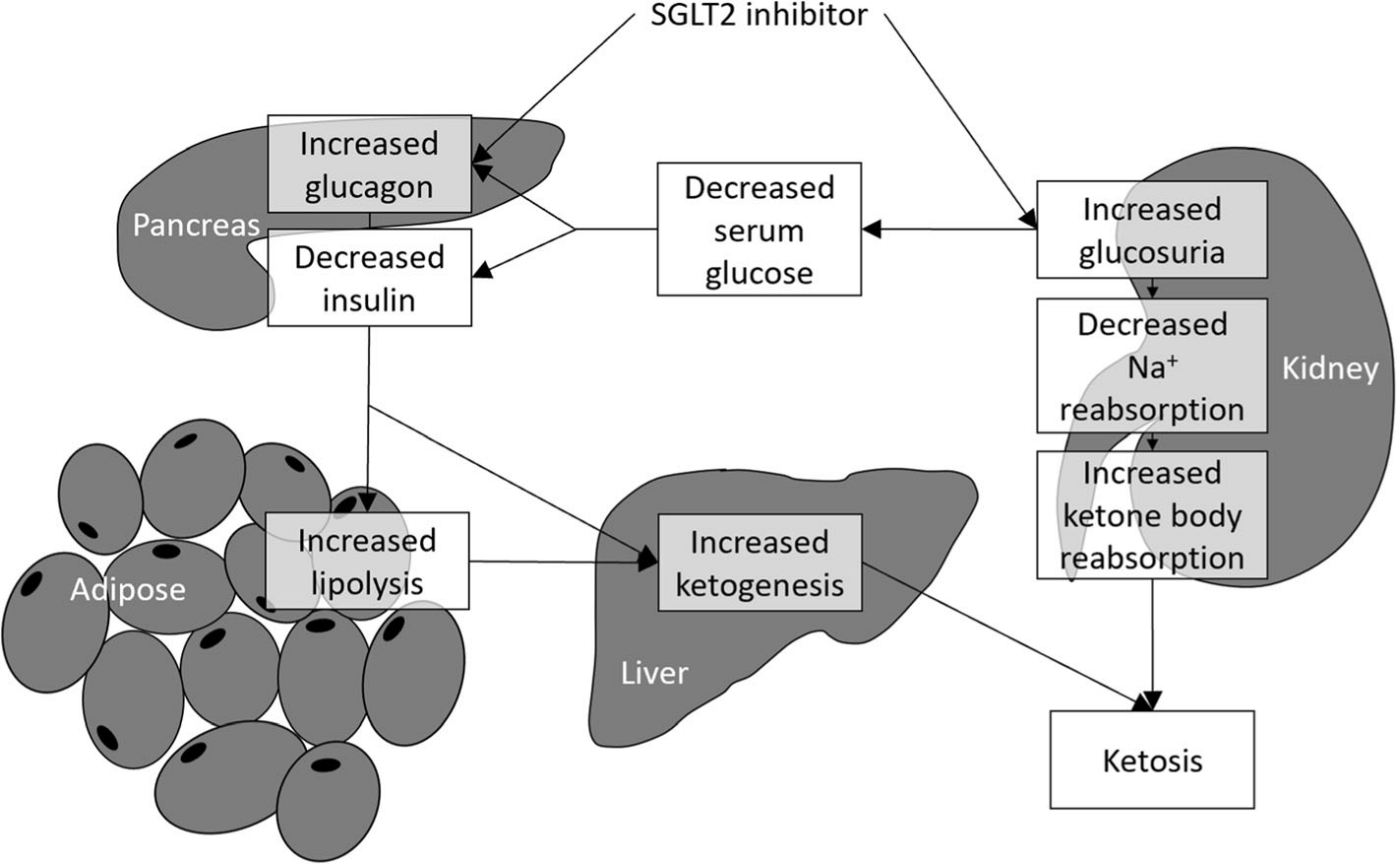
The possible mechanism of euglycemic diabetic ketoacidosis caused by SGLT2 inhibitor use

The possible mechanisms by which SGLT2 inhibitors cause EDKA are illustrated in Fig. 1. SGLT2 inhibitors decrease serum glucose primarily by increasing glucosuria in the kidney. They also appear to directly stimulate release of glucagon from the pancreas [14]. The glucosuria leads to decreased sodium reabsorption in the kidney, which in turn leads to increased ketone body reabsorption. The glucosuria results in lower serum glucose values which decreases insulin release and increases glucagon release from the pancreas. The increased glucagon-to-insulin ratio results in increased lipolysis and fatty acid oxidation and ketone production by the liver.

DKA in the setting of SGLT2 inhibitor use occurs in patients with type 1 DM with an incidence of 9.4% [15]. It is much less common when used in type 2 DM, with an incidence less than 0.2% [16, 17]. Several small case series and meta-analyses have reported on the characteristics of DKA associated with SGLT2 inhibitor use [10, 17–24]. DKA occurring in the setting of SGLT2 inhibitor use typically presents with minimal hyperglycemia, resulting in a delay of the appropriate diagnosis in over 50% of cases [18]. Reported median glucose values in these cases range from 211 to 328 mg/dL [20, 22, 23].

As with our patient, most patients presenting with DKA while taking a SGLT2 inhibitor will do so within 2 months of starting the medication [18, 19, 23]. Most patients with DKA while taking a SGLT2 inhibitor will have a precipitating event, with dehydration, infection, surgery, and changes in insulin dose being commonly reported. Other reported precipitating factors are listed in Table 1 [18, 20–23]. Symptoms of DKA associated with SGLT2 inhibitor use are similar to DKA in general, with nausea, vomiting, and abdominal pain most commonly reported [20, 22]. Patients with EDKA in particular may not manifest symptoms of dehydration to the degree of other DKA patients, given the lack of hyperglycemia [25, 26]. A recent US FDA review of adverse events associated with SGLT2 inhibitor use reported a fatality rate of 1.54%, as compared with 0.4% for all DKA cases [19, 27].

**Table 1:** Common precipitants of diabetic ketoacidosis while taking a SGLT2 inhibitor

Vomiting	
Dehydration	
Discontinuation or reduction of insulin dose	
Discontinuation of an oral insulin secretagogue	
Surgery	
Viral or bacterial infection	
Fasting or reduction of caloric intake	
Excessive ethanol use	

EDKA associated with SGLT2 inhibitor use should be treated in a similar fashion as other types of DKA, but with consideration of the lack of hyperglycemia. Initial treatment is directed towards volume resuscitation with isotonic saline. The initial fluid replacement should be followed by continuous intravenous insulin infusion at a rate of 0.02–0.05 units/kg/h [3]. Dextrose-containing fluids should be started along with the insulin infusion to avoid hypoglycemia, with a target serum glucose level of 150–200 mg/dL. Serum electrolytes and glucose levels should be monitored closely during the treatment course. Resolution of EDKA is identified by the presence of two of the following: a serum bicarbonate level ≥ 15 mmol/L, an anion gap ≤ 12 mmol/L, or a venous pH > 7.3 [3].

## Conclusions

The SGLT2 inhibitors are a new class of anti-hyperglycemic medications used in the treatment of DM. Their use has been associated with an increased risk of DKA. Many of the cases of DKA associated with SGLT2 inhibitor use will present with normal or minimally elevated serum glucose levels, and this frequently leads to a delay in diagnosis. The possibility of EDKA must be kept in mind when evaluating a patient with an unexplained metabolic acidosis while taking a SGLT2 inhibitor.

## Data Availability

Data sharing is not applicable to this article as no data sets were generated or analyzed during the current study.
